# Rhesus Monkeys See Who They Hear: Spontaneous Cross-Modal Memory for Familiar Conspecifics

**DOI:** 10.1371/journal.pone.0023345

**Published:** 2011-08-24

**Authors:** Ikuma Adachi, Robert R. Hampton

**Affiliations:** Yerkes National Primate Research Center, Atlanta, Georgia, United States of America; McMaster University, Canada

## Abstract

Rhesus monkeys gather much of their knowledge of the social world through visual input and may preferentially represent this knowledge in the visual modality. Recognition of familiar faces is clearly advantageous, and the flexibility and utility of primate social memory would be greatly enhanced if visual memories could be accessed cross-modally either by visual or auditory stimulation. Such cross-modal access to visual memory would facilitate flexible retrieval of the knowledge necessary for adaptive social behavior. We tested whether rhesus monkeys have cross-modal access to visual memory for familiar conspecifics using a delayed matching-to-sample procedure. Monkeys learned visual matching of video clips of familiar individuals to photographs of those individuals, and generalized performance to novel videos. In crossmodal probe trials, coo-calls were played during the memory interval. The calls were either from the monkey just seen in the sample video clip or from a different familiar monkey. Even though the monkeys were trained exclusively in visual matching, the calls influenced choice by causing an increase in the proportion of errors to the picture of the monkey whose voice was heard on incongruent trials. This result demonstrates spontaneous cross-modal recognition. It also shows that viewing videos of familiar monkeys activates naturally formed memories of real monkeys, validating the use of video stimuli in studies of social cognition in monkeys.

## Introduction

Many primate species have complex social repertoires that require individual recognition [1]–[7]. Field studies show that nonhuman primates recognize social objects and social events [5], [7]–[12], but field studies cannot address most questions about how nonhuman primates acquire complex social knowledge, or how this knowledge is represented in the brain. Controlled laboratory tests, in which learning is experimentally manipulated, are required to address these important questions in social cognition. To date, few such experimental studies of social recognition have been conducted; far more effort has been devoted to understanding how primates perceive, process, and remember nonsocial stimuli (see a recent review [13]).

The ability to keep track of the social relations of conspecifics is critical for survival in many species [5], [14] and individual recognition is a fundamental cognitive requirement for such mental tracking of the social environment. In primates, visual perception, especially of the face, is probably the most important source of information for identifying others [15]. Monkeys and apes do discriminate and identify specific faces (e.g. discrimination: [16]–[22], “identification” using symbols: [23], [24]), and recent studies have begun to characterize the underlying perceptual mechanisms for face recognition in nonhuman primates [25]–[29].

Most studies of individual recognition in monkeys have used still image stimuli, but social agents move. Dynamic social agents cannot be inspected in detail like still images, and the behavior of social agents has the potential to overshadow processing of physical features useful in individual identification. To understand natural social cognition it is therefore important to study how nonhumans extract information about dynamic social agents. Playback experiments conducted in the field demonstrate that monkeys recognize the dominance rank of other animals [30]. These findings motivated further study under more controlled conditions with captive animals. Rhesus monkey subjects learned to select dominant stimulus monkeys in video clips of both real dominance interactions [31] and digitally edited artificial dominance interactions [32]. Use of artificial social interactions in the latter work allowed random assignment of stimulus monkeys to ranks in an artificial hierarchy, thus controlling for non-behavioral cues that might indicate dominance. Subject monkeys rapidly learned to select the dominant monkey in these artificial social interactions, but only weakly transferred performance to probe videos containing no behavioral dominance information. These results show that monkeys “read” the behavior in the videos very effectively, but may have remembered little about the identities of the unfamiliar monkeys depicted. Because this study used unfamiliar stimulus monkeys, it is not possible to directly test whether subject monkeys treated the videos as valid representations of real world individuals and their dominance relations. The current study determined whether monkeys perceive videos as depicting actual monkeys, and whether they can extract identifying information from dynamic video displays.

Audition is also important for identifying others, particularly when distance or occluding objects render vision ineffective. Playback experiments conducted in the field confirm that monkeys discriminate voices of their group members and attribute them to the calling individual [8], [10], [11]. For instance, adult female rhesus macaques are more responsive to the contact calls of adult female kin than to those of unrelated females in the group [33] and vervet monkeys recognize third party kin relations on the basis of voice alone [30], [34].

Recognition of individuals by appearance, especially face, and by voice is clearly advantageous, but the flexibility and utility of primate social memory would be greatly enhanced if visual and auditory memories could be accessed cross-modally by stimulation in either modality [35]. For instance, human representations of individuals appear to integrate visual and auditory features [36], as evident when we visualize the speaker on the other end of a phone call. Field playback experiments in which subject hear the call of a particular individual, and then demonstrate that they expect to see that individual or an associated individual, provide some of the best evidence that cross-modal processing of individual identity is a central part of primate social life. However, few laboratory studies have tested for such cross-modal representations in primates. In the first study of its kind, Guinea baboons (*Papio papio*), were trained to discriminate between human and baboon vocalizations and were then given probe trials in which either a human or a baboon photo was presented just before a vocalization [37]. Priming with a photo matching the vocalizing species shortened response time in one of the two baboons, suggesting that one subject had formed arbitrary associations between species typical sounds and visual appearance. The priming image may have activated corresponding auditory representations in this one baboon, leading to facilitation of processing the subsequent auditory stimulus. More recent studies used a cross-modal version of the expectancy violation procedure pioneered by Adachi and his colleague [38]. Human or Japanese macaque vocalizations were played repeatedly through two speakers, followed immediately by an image that either matched (congruent condition) or mismatched (incongruent condition) the auditory stimulus. Subjects looked longer at the image in the incongruent condition, indicating that they had formed the expectation of seeing an image consistent with the vocalization [38], [39]. Other nonhumans also appear to form cross-modal representations of “species” that they are familiar with, for example human caretakers (dogs: [40]), familiar conspecifics (grey-cheeked mangabeys (*Lophocebus albigena*): [41]; horses: [42]), and familiar conspecifics and humans (rhesus monkeys tested by preferential looking procedure; [43]). These procedures that measure the spontaneous looking behaviour of subjects are useful for comparisons across species because they require no training of the subjects. However, they are limited by the need for many subjects due to variability in looking time, and by the fact that animals cannot be tested repeatedly due to habituation. Detailed study of the nature of the animals' mental representations will require additional techniques.

In the present study, we focused on two aspects of individual recognition in rhesus monkeys. First, we tested whether they could recognize dynamic images of familiar individuals in video clips. Second, we tested whether they had formed cross-modal representations of those familiar individuals through experience outside of our experiment, and whether those representations were activated by seeing videos. We used a delayed matching-to-sample procedure in which subjects were trained to visually match a video clip of a familiar individual to a photograph of that individual presented among 4 distracter images of other familiar monkeys. Auditory stimuli were never used during training. In later probe trials, a voice, either matching the sample video clip (congruent trials) or not (incongruent trials), was played during a memory interval. We assessed spontaneous cross-modal recognition by determining the extent to which: 1) monkeys were more accurate on congruent compared to incongruent trials, and, 2) errors made on incongruent trials were to the image of the monkey whose voice was played during the memory interval. Discrimination of familiar conspecifics in video clips could, of course, be accomplished without recognizing the stimuli as familiar conspecifics. Monkeys might instead learn that specific properties of the videos occasion specific test responses. However, spontaneous cross modal recognition could occur only if the subject monkeys recognized the individuals in the videos as those they live with. If monkeys did not detect a correspondence between the videos and the real monkeys, the untrained vocalizations could not systematically affect choice behavior.

## Experiment 1A

### Method

#### Subjects

Subjects were five 4-year-old male rhesus monkeys (*Macaca mulatta*) that had been raised in large semi-natural social groups at the Yerkes Primate Center field station up to about 2.5 years of age. Each monkey shared a cage with a single compatible companion. Monkeys had visual and auditory contact with additional monkeys living in the same room. The Yerkes National Primate Research Center is fully accredited by the American Association for Accreditation of Laboratory Animal Care. Animals were cared for and used in accord with the Guide for the Care and Use of Laboratory Animals published by the National Academy Press and in a manner consistent with the recommendations of the Weatherall Report on the use of non-human primates in research. The procedures used in this study were approved by Emory University's Institutional Animal Care and Use Committee (protocols 222-2004Y and 206-2007Y). Among the steps taken to maximize welfare and minimize suffering were the following. The monkeys were provided with enrichment according to Yerkes policy to maximize psychological well-being through visual and social stimulation. The investigators used positive reinforcement training techniques to ensure calm interactions with the monkeys, and for cognitive testing. The majority of cognitive testing was conducted in the home cage environment in the presence of established social companions. No potentially painful procedures were used in these studies.

#### Apparatus

Monkeys were trained in their home cage using an apparatus consisting of a 15-inch color LCD monitor with a capacitance touch sensor (3 M, St. Paul, MN), two food dispensers (Med Associates, St. Albans, VT; one delivered banana flavored monkey pellets and the other miniature chocolate candies), and a loudspeaker, all of which attached to the front of the cage housing the monkey. Testing was controlled by a personal computer with custom software written using Presentation© (Neurobehavioral Systems, Albany, CA).

#### Stimuli

Each subject monkey was assigned 160 silent 5 second videos clips (640×480 pixels, 30 fps) consisting of 32 videos of his cagemate, and 32 videos from each of four other familiar monkeys that lived in the same room in auditory and visual contact with the subject monkey. Twenty six video clips from each set of 32 were used in a series of training and testing cycles, and six were used for a final transfer test. All training videos showed individual stimulus monkeys in the same cage and were generated under identical lighting conditions. The six video clips in each set used for the final transfer test were filmed from a hole on the backside of each monkey's home cage and thus depicted substantially different views of the monkeys from those in the training videos. Face-on still pictures (200×200 pixels) of the same stimulus monkeys shown in the videos were used as choice stimuli.

#### Procedure

**Figure 1:**
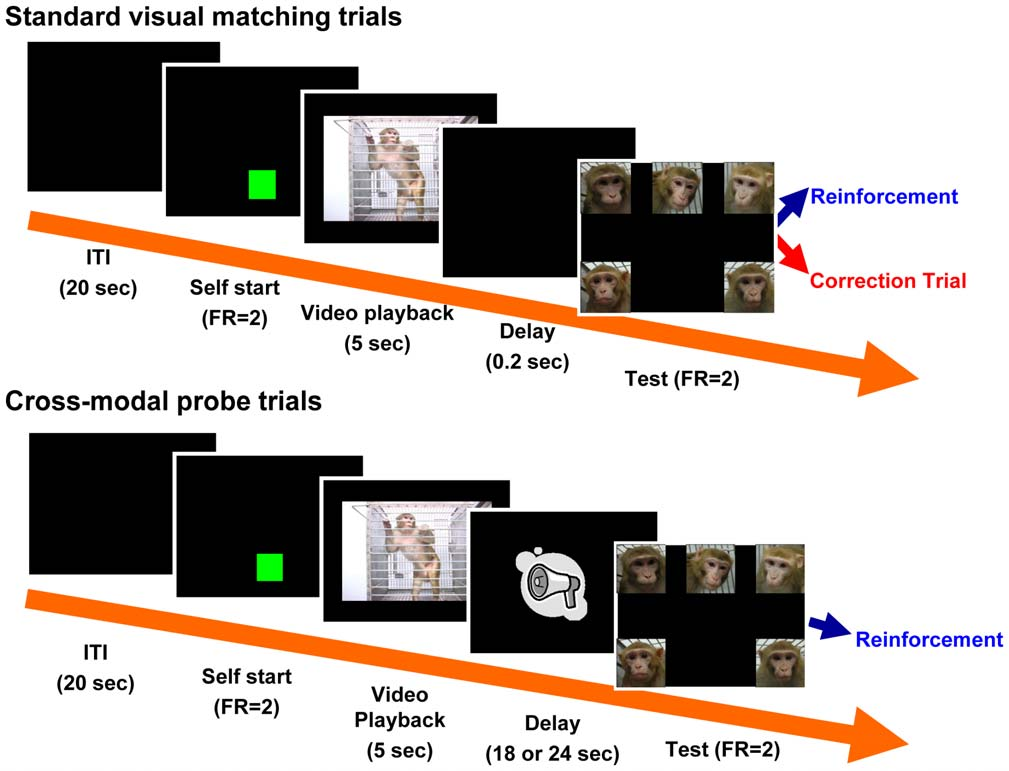
This figure shows schematic diagrams of the visual matching-to-sample tasks used in the current study. The top panel depicts the task used in Experiment 1 and as baseline in Experiment 2 (Standard visual matching trials). The lower panel depicts test trials in Experiment 2 (Cross-modal probe trials).

During training and testing monkeys remained in the homecage, but pairs were separated by panels with holes cut in them such that social interaction was possible but monkeys could only reach the computer screen in their own cage. Figure 1 (top row) depicts the delayed matching-to-sample task used. Each trial started when the subjects touched a green rectangle twice. A 5 second video clip of one stimulus monkey then played in the center of the monitor as a sample stimulus. After the video ended, the last frame remained on the monitor. Two touches on the last frame extinguished it and resulted in the appearance of still pictures of the five stimulus monkeys, one located in each of the four corners and one in the middle of the top of the monitor. The locations of these five pictures were randomly determined on each trial. Touching the choice stimulus that corresponded to the sample was reinforced by the automatic delivery of food, whereas touching the incorrect comparison stimulus was followed by a half second time-out and a correction procedure. In the correction procedure the trial was repeated up to three times. If the monkey erred in all of these trials, a final trial was given in which only the correct choice appeared at test.

Monkeys were trained and tested in this visual matching-to-sample task in five phases. In Phase 1, subject monkeys were trained with two video clips from each stimulus monkey (10 videos total). After performing at above 90% correct in two consecutive sessions, the monkeys proceeded to Phase 2, in which they received six new video clips from each stimulus monkey (30 new videos total) in addition to the two trained clips. Every time monkeys reached the criterion of 90% or better in two consecutive sessions, they proceeded to the next phase with six new video clips from each stimulus monkey. In the first session of each of the phases 2–5, each new video clip was presented only once and we did not use the correction procedure. We therefore measured performance in the very first exposure to each of the 30 new videos in the new set of test stimuli in these initial sessions. At the end of Phase 5, monkeys were therefore required to perform above 90% correct with 26 videos from each stimulus monkey (a total of 130 videos).

In the final transfer test, monkeys received two test sessions in which the 30 transfer videos (6 from each stimulus monkey) that had been filmed through a hole in the back of the homecage were interspersed among 130 control trials consisting of all the video clips from phases 1 through 5 To prevent any new learning in these final generalization test trials, monkeys were always rewarded, irrespective of the accuracy of their choice.

### Results

**Figure 2:**
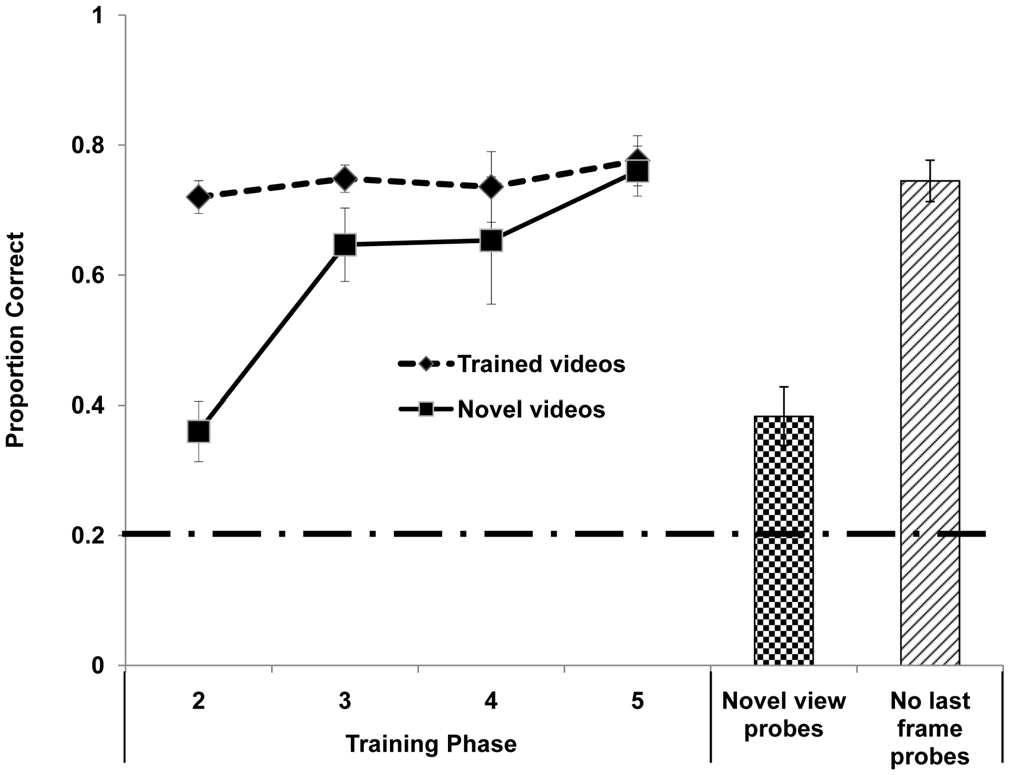
Line Graph: Generalization to novel videos in Experiment 1A. The uppermost line (diamond symbols, dashed line) represents trials with all familiar videos from previous phases of training, only during test sessions in which new videos were introduced. The lower data line (square symbols, solid line) represents performance on the first exposure to newly introduced videos. The bottom dashed line indicates accuracy expected by chance. Bars: Generalization to test trials in Experiment 1A and Experiment 1B. The bar with checker pattern represents performance in probe trials with novel videos depicting stimulus monkeys from the novel perspective of the back of a housing cage, during a single test session (Experiment 1A). The hatched bar represents performance in probe trials in which the screen went black and choice images appeared immediately after the video ended.

Monkeys learned to select the comparison still image corresponding to the sample videos. Accuracy on the first exposure to novel sets of videos improved with successive introductions until performance with novel videos did not differ from that with highly familiar videos (Figure 2; t-tests comparing familiar with novel videos: 2nd phase: t(4) = 6.175, *p*<.01, 3rd phase: t(4) = 1.618, n.s., 4th phase: t(4) = 1.199, n.s., 5th phase: t(4) = .734, n.s.). In the transfer test with videos collected from an entirely new perspective and in a different context, monkeys transferred matching performance to the novel test videos significantly better than expected by chance (Figure 2; t(4) = 8.572, p<0.01) though performance was significantly lower than with the novel stimulus set used in phase 5 (t(4) = −7.387, p<0.01).

## Experiment 1B

In Experiment 1A, the last frame of each clip remained on the screen until subjects touched it. This procedure was used to ensure that monkeys studied the sample before responding. However, monkeys may have used the last frames to solve the task, rather than the dynamic information in the videos. In Experiment 1B, we tested whether monkeys could solve the task using the movie in real time, without the last frames remaining on the screen. The same apparatus used in Experiment 1A was used again. The procedure differed from Experiment 1A only in that the screen went black and the choice stimuli were presented immediately after the videos ended.

### Results

Monkeys transferred matching performance to the test trials without the last frame frozen (Figure 2, rightmost bar). All five subjects continued to perform significantly better than expected by chance (each subject *p*<.001 by binomial test). Performance was numerically almost identical to that shown in the last block of testing with videos followed by a still frame. Subjects appear to have focused on the dynamic information in the videos rather than using the last still frame to identify the correct choice at test.

## Experiment 2

Monkeys accurately matched short videos to still images of familiar monkeys in Experiment 1, and generalized this performance on the first exposures to never before seen videos. Even when we tested our subjects with videos from substantially different views and context in a final transfer test, they showed significant transfer of matching performance. These results show that monkeys extracted invariant features from a subset of videos that allowed them to generalize selection of the appropriate still image across the considerable variation in the sample videos. While such successful generalization suggests that the monkeys recognize the familiar monkeys depicted in the videos as those they live with, it is possible that performance is based strictly on similarity among the videos, with no reference to memories of the familiar monkeys formed outside the context of the experiment. Indeed, many experiments show that animals learn to accurately classify images into categories even when they have had no real world experience with the individual items being classified or with the categories (e.g. laboratory pigeons appropriately classify images of cats, cars, flowers, and chairs [44]).

In Experiment 2, we tested whether viewing videos of familiar monkeys activated memories of those monkeys that were formed during real social interactions outside of the context of our experiment. Because our monkeys were trained exclusively with images in Experiment 1, they have no basis in our training for mapping monkey voices to the videos or still images used in these experiments. Only natural experience with vocalizations and faces in the colony room could allow the monkeys to integrate visual and auditory properties of the stimulus monkeys. We reasoned that if hearing monkey vocalizations systematically biased test performance in our visual matching task, then performance must be mediated in part by cross-modal memories of the real monkeys formed during social interactions in the colony room. We therefore tested whether the rhesus monkeys in our study have multimodal access to memories of familiar conspecifics.

### Subjects and apparatus

In order to use auditory stimuli, monkeys were removed from the home cage and isolated in a sound attenuating booth for testing sessions. Isolation was necessary because coo calls played in the housing room elicited many coo calls from other animals in the room. Because the other monkeys living in the room were the stimulus monkeys used in this study, calls from these monkeys, whether elicited or spontaneous, would interfere with testing. All previous testing with these monkeys had been conducted in the home cage in a very familiar social context. Perhaps because of this extensive experience with testing in the home cage, we had difficulty adapting the monkeys to the visual and auditory social isolation of the sound attenuating booths. Despite many weeks of daily experience in the booths, we were only able to adapt two of the five monkeys sufficiently well to permit participation in this study (M1 and M2).

#### Stimuli

Coo calls, which are a contact call known to carry identity information (e.g. [45]), were recorded from each stimulus monkey using a digital audio recorder (Marantz PMD660) and a “shotgun” microphone (Sennheiser ME 66). Recordings were converted to WAV format sampled at 44.1 KHz and 16-bit resolution. The duration of each vocalization was approximately 750 ms.

#### Procedure

Before beginning cross-modal tests, the performance of monkeys was titrated to approximately 60% accuracy by gradually increasing the delay between sample and test. This was done to ensure that monkeys would make a sufficient number of mistakes for analysis of errors and that performance could both increase and decrease as a result of hearing vocalizations. Titration was done in the home cage and later confirmed in the testing booth. At the conclusion of titration, the delay between the end of the sample videos and the appearance of the still choice images for the test trials was 18 s for M1 and 24 s for M2.

Subjects received 15 test sessions, each consisting of 30 all-reinforced cross-modal probe trials interspersed among 160 baseline vision only trials identical to those used during training. For the baseline trials, the delay lengths were randomized among .5, 4.0, 8.0, and either 18.0 (for M1) or 24.0 sec (for M2). This distribution of delay intervals was intended to maintain motivation and to prevent monkeys from predicting which trials were probe trials. Cross-modal probe trials began the same way as normal trials, with presentation of a 5 second video clip, followed by two touches to the last frame by the subject monkey. Immediately after the monkeys touched the last frame of the video, a vocalization was played. The delay of 18 or 24 seconds ensued, followed by presentation of the 5 still images of monkeys used in all previous testing. For each session, one of the 32 clips from each stimulus monkey was used as a sample stimulus in probe trials. We presented three test conditions in each session. In the congruent condition, a vocalization from the same monkey seen in the sample video was played just after the sample stimulus disappeared (5 trials, one from each stimulus monkey). In the incongruent condition, a vocalization from a stimulus monkey other than the one seen during the sample phase of the trial was played (20 trials, 4 from each stimulus monkey, thereby pairing each stimulus monkey with each possible incongruent vocalization). In the control condition no vocalization was played but the same delay was used as on the other test trials (5 trials, one from each stimulus monkey; see Figure 1B). At the conclusion of the 15 sessions of crossmodal testing we therefore had 75 congruent probe trials, 300 incongruent probe trials, and 75 control trials from each of the two subject monkeys. Monkeys were never trained to use the vocalizations to guide their test response; to prevent learning during the probe trials all responses on these trials were rewarded whether correct or not.

We hypothesized that if monkeys have cross-modal representations of familiar monkeys, hearing a vocalization would activate a representation of the vocalizing monkey and that representation would facilitate (Congruent trials) or interfere with (Incongruent trials) visual matching accuracy. We also assessed interference by determining whether errors on Incongruent trials were made more often than expected by chance to the image of the monkey whose voice was heard during the memory interval.

### Results

**Figure 3:**
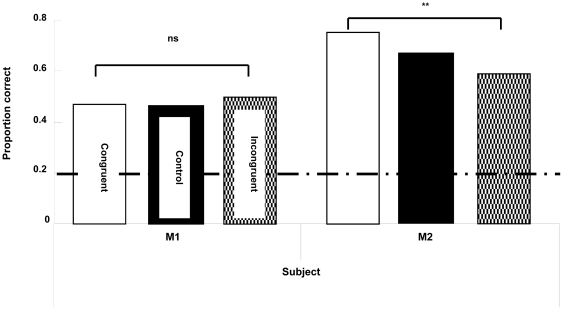
Proportion of correct choices on trials with a vocalization from the monkey seen in the sample video (Congruent), without a vocalization (Control), or with a vocalization from a monkey other than the one seen in the sample video (Incongruent) in Experiment 2. Accuracy expected by chance is 0.2.

**Figure 4:**
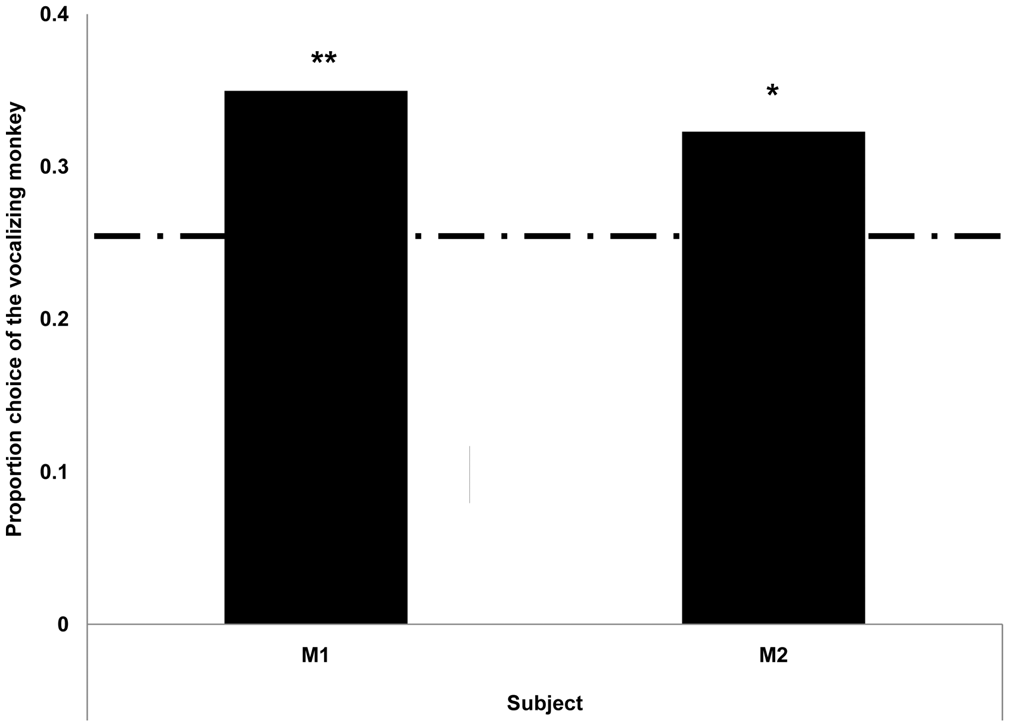
Proportion of errors made by selecting the vocalizing monkey in incongruent trials from Experiment 2. The dashed line represents the proportion of choices expected by chance.

In all test conditions, both monkeys were more accurate than expected by chance (binomial tests, *p*<.01; Figure 3). To examine the effect of the vocalizations that were played during the memory interval, we conducted paired t-tests for each combination of the three conditions in each monkey, with alpha set at 0.0167 to control for multiple comparisons. For M2, performance in the Congruent condition was significantly higher than in the Incongruent condition, but neither condition differed from the Control condition (Incongruent vs. Congruent: *t*(14) = 3.263, *p* = .003; Congruent vs Control: *t* (14) = .501, n.s.; Incongruent vs Control: *t* (14) = −1.640, n.s.). For M1, there were no significant differences in performance among the three conditions (Congruent vs Incongruent: *t* (14) = −.486, n.s.; Congruent vs Control: *t* (14) = .164, n.s.; Incongruent vs Control: *t*(14) = 1.097, n.s.). We also analyzed choice behavior on trials on which monkeys committed an error. Both monkeys picked the image of the vocalizing monkey more often than expected by chance (25%) when committing an error (Figure 4; binomial tests: M1, *p*<.01; M2, *p*<.05).

Monkeys did not perform exactly at the targeted level of 60% correct in this final stage of testing. It is not clear whether the differences from the titrated levels are experimental noise, an effect of the surprising recorded monkey calls on attention or motivation, or other changes resulting from continued testing. In any case, these shifts in accuracy on control trials do not affect our ability to interpret the results of the present experiment because performance in probe trials is compared to concurrently run control trials.

## Discussion

In Experiment 1A monkeys initially learned to match a set of 10 videos to still pictures of 5 familiar monkeys. Generalization to new videos improved greatly following experience with more videos, until performance with novel videos was indistinguishable from that with familiar videos. Subjects continued to perform well above the level expected by chance in very challenging generalization tests with videos collected from a new view and in a different context (Figure 2). While the significant generalization observed clearly demonstrates that the subject monkeys did not memorize videos or use low level perceptual processes to identify stimulus monkeys, accuracy in these very challenging generalization tests was lower than accuracy in less challenging tests. Lower performance in these tests could result from several factors, including the somewhat poorer quality of these videos, which were taken through a small hole in the cage, and the possibility that the attention of monkeys is drawn away from the stimulus monkey shown in the video by the many new objects also visible for the first time in the novel view videos.

Experiment 1B, showed that monkeys did not depend on the last still frame of videos to guide their matching performance in Experiment 1A. They continued to perform accurately even though the screen went black immediately after the video ended, meaning that the final frame was on screen for just 1/30 seconds. This finding does not rule out use of information in the last frame, but does show that this frame need not be still in order for monkeys to perform accurately. Thus it is likely that subject monkeys used the dynamic content of the videos to identify the stimulus monkeys, rather than any single brief video frame. These successful generalizations suggest that the monkeys recognized the individuals depicted across the full set of videos. However, it is possible that the monkeys based their test choices on superficial similarities shared by all the videos of a given stimulus monkey, rather than by reference to representations of those monkeys formed during live interactions with them in the colony room. Thus, the data from Experiment 1 do not conclusively show that monkeys perceived the videos as depictions of monkeys they know.

Despite the fact that we had trained monkeys exclusively to follow a visual matching rule in Experiment 1, hearing vocalizations systematically biased choice behavior in Experiment 2. When monkeys heard the voice of a different monkey than the one they saw in a sample video clip, both of the subjects made errors by selecting the image of the owner of the voice more often than expected by chance. This effect was significant for each monkey but was not large. The small size of the effect is consistent with the fact that the monkeys had never been trained to use auditory information to guide choices at test. Training in the social context of the housing room may, indeed, have taught monkeys to actively ignore vocalizations they heard during testing because they were not relevant to the rewarded visual matching task. The fact that the monkeys showed significant effects from the auditory information at all is remarkable and indicates spontaneous cross modal recognition. One of the two subjects also showed better performance in Congruent trials (on which video and voice matched) than in Incongruent trials (on which video and voice did not match). Together, these results indicate that the monkeys had cross-modal representations of the familiar monkeys depicted in the videos. Hearing the voices of these monkeys crossmodally activated visual representations of them, and these representations sometimes superseded the representations activated by seeing the sample video. Apparently, sometimes the monkeys could not discriminate between active visual representations that resulted from seeing a video and those resulting from hearing a voice.

It is important to note that the monkeys had not been trained to associate the voices and visual information in these experiments. They were trained to focus exclusively on visual information. However, the presentation of vocalizations impacted visual-visual matching performance. The cross-modal representations demonstrated here must have been acquired in natural social interactions in the colony room. This study therefore shows that video stimuli used in laboratory based cognitive tests can activate memories formed during natural social encounters. The interaction of auditory and visual information we observed could only occur if the monkeys regarded the videos as depicting familiar monkeys. These findings set the stage for further ecologically valid laboratory studies of social cognition using videos.

In the current study we found that coo calls evoked visual information in subject monkeys. In future studies, it will be of interest to test whether other call types or visual information similarly activate common representations. Such studies will allow us to assess the function of these signals in primate social life in well controlled experimental studies.

Monkeys likely discriminate others based on various other attributes, in addition to identity. For instance, kin-recognition and sex categorization must play a fundamental role in reproductive success in primates, so primates should be keenly attuned to information specifying kinship and sex. For example, previous studies reported that some primate species can detect kinship visually [46] and vocally [30], [47]. More recently, it is reported that body parts with conspicuous sexual features (male scrotum or female nipples) facilitate discrimination of gender in Japanese monkeys [48]. Such studies have been limited to the visual modality or auditory modality only and can potentially be explained by basic perceptual level discrimination, without reference to any more abstract concept of sex or kinship. The general approach used here could be extended to test for the existence abstract social concepts such as “sex” or “kin.” Only conceptual representations that exist at a level more abstract than perception would be spontaneously activated crossmodally.

An important aspect of cross-modal representation awaiting clarification is whether there is any preferred or privileged direction of cross-modal activation, or a dominant modality of representation. Monkeys showed cross-modal activation both in the visual to auditory direction [37] and the auditory to visual direction (current study). While only one of two baboons in the former study showed evidence of visual to auditory activation, both subjects in our study showed evidence of auditory to visual activation. Animals that rely on vision as the primary perceptual modality for the control of behavior, may show visual dominance in mental representation, favoring visual representations accessible by other modalities rather than representations in those other modalities per se. To examine this issue directly, future studies might compare visually dominant with auditory dominant species for the ease with which auditory and visual stimulation activate representations in the other modality.

These experiments show that video stimuli elicit sophisticated information processing sufficient for individual recognition in rhesus monkeys. In conjunction with other recent findings that suggest ecologically relevant processing of videos by monkeys viewing faces [28], [29], and assessing social behavior [31], [32], these results encourage increased use of carefully controlled video stimuli in studies of primate social cognition. Spontaneous cross-modal activation of visual representations of familiar monkeys by their vocalizations unequivocally demonstrates that our subject monkeys regarded video stimuli as depicting monkeys they knew. Whether they saw them in videos or heard their voices, memories of the monkeys they knew were activated.
